# Impact of spontaneous liposome modification with phospholipid polymer-lipid conjugates on protein interactions

**DOI:** 10.1080/14686996.2022.2146466

**Published:** 2022-12-08

**Authors:** Haruna Suzuki, Anna Adler, Tianwei Huang, Akiko Kuramochi, Yoshiro Ohba, Yuya Sato, Naoko Nakamura, Vivek Anand Manivel, Kristina N Ekdahl, Bo Nilsson, Kazuhiko Ishihara, Yuji Teramura

**Affiliations:** a Department of Systems Engineering and Science, Graduate School of Engineering and Science, Shibaura Institute of Technology, Saitama, Japan; b Department of Immunology, Genetics and Pathology (IGP), Uppsala University, Uppsala, Sweden; c Department of Bioengineering, School of Engineering, The University of Tokyo, Tokyo, Japan; d Cellular and Molecular Biotechnology Research Institute (CMB), National Institute of Advanced Industrial Science and Technology (AIST), Ibaraki, Japan; e Department of Bioscience and Engineering, College of Systems Engineering and Science, Shibaura Institute of Technology, Saitama, Japan; f Linnaeus Center of Biomaterials Chemistry, Linnaeus University, Kalmar, Sweden; g Division of Materials and Manufacturing Science, Graduate School of Engineering, Osaka University, Osaka, Japan; h Master’s/Doctoral Program in Life Science Innovation (T-LSI), University of Tsukuba, Ibaraki, Japan

**Keywords:** 2-Methacryloyloxyethyl phosphorylcholine (MPC) polymer, liposomes, surface modification, anti-PEG antibody

## Abstract

Liposome surface coating has been studied to avoid the immunological responses caused by the complement system, and alternative materials to poly(ethylene glycol) (PEG) have been explored recently since the production of anti-PEG IgM antibodies has been found in humans. We previously reported a liposome coating with poly(2-methacryloyloxyethyl phosphorylcholine) (poly(MPC))-conjugated lipids (PMPC-lipids) and demonstrated its protective effect on blood protein interactions. Here, we attempted to modify the liposome surface by exogenously adding PMPC-lipids, which were spontaneously incorporated into the outer membrane via hydrophobic interactions. The polymerization degree of the PMPC segment was regulated from 10 to 100. The incorporated ratio of PMPC-lipid increased with a decrease in the degree of PMPC polymerization. Due to surface modification with PMPC-lipids, increase in the length of the PMPC-chain increased the size of the liposomes. The modified liposomes were kept stable for 14 d in terms of their size, polydispersity, and surface properties, where approximately 70% of PMPC-lipids were incorporated into the liposome surface. We demonstrated that liposome surface modification with PMPC-lipids can inhibit protein adsorption when exposed to serum, regardless of the degree of polymerization of PMPC. In addition, the PMPC-lipid modified surface was not recognized by the anti-PEG IgM antibody, whereas PEG-lipid was recognized by the antibody. Thus, we successfully fabricated an inert liposome surface via spontaneous modification with PMPC-lipids, where only the outer bilayer surface was modified. This technique can be available for full loading of water-soluble active pharmaceutical ingredient inside the modified liposome.

## GRAPHICAL ABSTRACT

**Figure:**
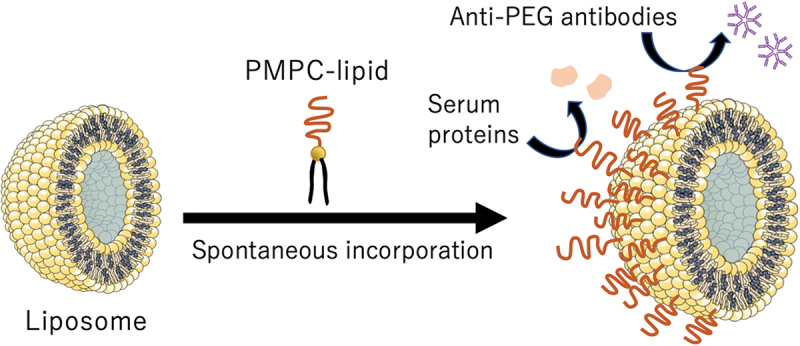

## 1. Introduction

Liposomes have gained much interest as promising drug delivery systems since their discovery in 1965 by Bangham et al. [1]. Liposomes are small artificial lipid vesicles composed of phospholipids with or without cholesterol. Phospholipids and cholesterol are amphiphilic molecules that orient themselves in the most energetically favorable structure in aqueous solutions; thus, they spontaneously form spherical vesicles with single or multiple lipid bilayer membrane(s), known as liposomes [2]. Liposomes can be used to carry a broad range of molecules, as they can encapsulate hydrophilic compounds in their aqueous core, while the lipophilic compounds can be trapped within the lipid bilayer [3–7]. The encapsulated drugs are protected from premature degradation, thereby reducing the drug-induced damage to healthy tissues and minimizing systemic toxicity [2]. Currently, several United States Food and Drug Administration- and European Medicines Agency-approved liposomal drugs, such as Doxil and AmBisome, are being used in clinical settings [8,9]. Even though liposomes lack the highly complex and dynamic environment of the cell membrane, they are used in research to simulate simple cell membranes or to investigate lipid – protein interactions owing to their cell-like membrane structure [2].

Enhancing the systemic circulation time of liposomes is key to obtaining a successful liposome-based drug delivery system that can deliver encapsulated drugs to the site of interest, for example, to facilitate the accumulation of liposomes in tumors via the enhanced permeability and retention effect [10]. However, *in vivo* liposomes interact with biological surroundings, leading to non-specific adsorption of plasma proteins and rapid clearance of liposomes from the circulation, which is generally detected for nanoparticles [11–13]. Therefore, the surface modification of liposomes with poly(ethylene glycol) (PEG), a synthetic, flexible, uncharged, and water-soluble polymer, is the gold standard to reduce liposome – protein interactions [14]. Bound water molecules generate a hydration layer around the PEG chains, thus suppressing the non-specific protein adsorption on liposomes [15]. However, PEGylated liposomes are rapidly cleared from the circulation upon repeated injections due to the formation of anti-PEG antibodies via the accelerated blood clearance effect (ABC effect) [16], which was first described by Dams et al. [17]. Additionally, there have been reports on the generation of anti-PEG antibodies in healthy individuals, who have not previously been administered any PEGylated therapeutics, which may also contribute to the reduced circulation time of PEGylated liposomes [18]. Therefore, it is essential to develop effective alternatives to PEGylated liposomes. Our group has focused on poly(2-methacryloyloxyethyl phosphorylcholine (MPC)) (PMPC), which is used for coating clinical biomedical devices [19], and PMPC-conjugated lipids have been studied as alternative liposome coatings [20–23].

We have previously revealed that PMPC-lipids can be used to coat liposomes via ‘pre-modification’ [22,23], that is, by dissolving the PMPC-lipids together with other lipids in ethanol and generating a dry lipid film, which is subsequently hydrated with a buffer solution to fabricate PMPC-lipid-modified liposomes. By dissolving PMPC-lipids and other lipids in advance, PMPC-lipids are distributed both inside and outside the lipid bilayer membrane in liposomes, where PMPC layers are formed on both sides of the membrane. When we used a mixture of PMPC-lipid and lipids in advance for liposome preparation, the inner space was smaller because of the PMPC polymer layer. This is particularly pronounced when the size of the liposome is small (approximately 100 nm) because the polymer layer is approximately 10–20 nm in size. This influences the drug-loading capacity of small liposomes with a diameter of approximately 100 nm, as the size of the aqueous core is reduced.

In this study, we investigated whether PMPC-lipids could be used to coat liposomes via spontaneous modification driven by hydrophobic interactions, where PMPC-lipids were externally added to intact liposomes. The advantage of this surface modification is that the inner space of the liposome is fully available for loading water-soluble active pharmaceutical ingredients. We also studied the interactions between PMPC-lipids and liposomes, and the stability of the modified liposomes. Finally, we analyzed the interactions of the modified liposomes with plasma proteins and anti-PEG antibodies.

## 2. Materials and methods

### 2.1. Methods

Dichloromethane, 1-dodecanethiol, triethylamine, α-bromoisobutyryl bromide (BIBB), tris(2-pyridylmethyl) amine (TPMA), 1,6-diphenyl-1,3,5-hexatriene (DPH), 5(6)-carboxyfluorescein (CF), trizma base, MPC, albumin-fluorescein isothiocyanate conjugate (FITC-albumin), bovine serum albumin (BSA) and glycine were purchased from Sigma-Aldrich Chemical Co. (St. Louis, Missouri, USA), while 1,2-dipalmitoyl-sn-glycerol (DPG) was purchased from Bachem Holding AG (Bubendorf, Switzerland). 1,2-Dipalmitoyl-sn-glycero-3-phosphocholine (DPPC) and 1,2-dipalmitoyl-sn-glycero-3-phosphoethanolamine-N-[monomethoxypoly(ethylene glycol)(5000)] (PEG-lipid) were purchased from the NOF Corporation (Tokyo, Japan). Anti-PEG antibody (BSA and azide-free, 26A04) was purchased from Abcam (Cambridge, UK). Latex beads (aldehyde/sulfate latex) and the microBCA protein assay kit were purchased from Thermo Fisher Scientific (Waltham, Massachusetts, USA). Sodium dodecyl sulfate (SDS), distilled water, copper(II) bromide (CuBr2), L(+)-ascorbic acid, cholesterol, ethanol (99.5%), cholesterol quantification kit (T-Cho E), tetrahydrofuran (THF), sodium chloride (NaCl), ammonium molybdate tetrahydrate, sodium hydrogen sulfite, sodium sulfite, 1-amino-2-naphthol-4-sulfonic acid, hydrogen peroxide, sulfuric acid, hexane, 1,4-dioxane, diethyl ether, hydrochloric acid, magnesium sulfate, methanol, dichloromethane (super dehydrated), chloroform-D, methanol-D4, acrylamide, bisacrylamide, ammonium persulfate, N,N,N’,N’- tetramethylethylenediamine, and quick-CBB were purchased from FUJIFILM Wako Pure Chemical Corporation (Osaka, Japan). Phosphate-buffered saline (PBS) and fetal bovine serum (FBS) were purchased from Gibco (Waltham, Massachusetts, USA). A dialysis membrane (3.5 kDa, Spectra/Por) was purchased from Repligen Corporation (Waltham, Massachusetts, USA). The PL2070–0100 PEG calibration kit (PEG-10) was purchased from Agilent Technologies (Santa Clara, California, USA). Tris-buffered saline (TBS) was purchased from TAKARA BIO, Inc. (Shiga, Japan). Bromophenol blue, 2-mercaptoethanol, and glycerol were purchased from Nacalai Tesque Inc. (Kyoto, Japan).

### 2.2. Synthesis and analysis of PMPC-lipids

#### 2.2.1. Synthesis of PMPC-lipids

**Scheme 1.**
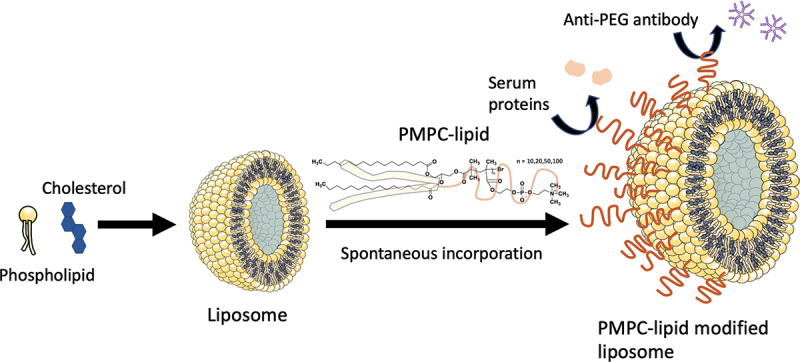
Schematic illustration of exogeneous liposome modification with poly(2-methacryloyloxyethyl phosphorylcholine) (polyMPC)-conjugated lipids (PMPC-lipids) and its influence on protein adsorption.

PMPC-lipids with polymerization degrees of 10, 20, 50, and 100 (3.7, 6.7, 16, and 30 kDa, respectively) were synthesized as reported by Adler et al. [22] (Scheme 1). These lipids are referred to as MPC10-lipid, MPC20-lipid, MPC50-lipid, and MPC100-lipid, respectively. Briefly, an atom-transfer radical polymerization (ATRP) initiator composed of DPG and BIBB was synthesized. DPG (400 mg) with two acyl chains of C16 length was dissolved in dichloromethane (4 mL) and triethylamine (85.4 mg) was added. BIBB (194 mg) was dissolved in dichloromethane (1 mL), followed by stepwise addition at room temperature and an overnight reaction. The molar ratio of DPG, triethylamine, and BIBB was [DPG]/[triethylamine]/[BIBB] = 1/1.2/1.2. Hexane (30 mL) was then added to the reaction solution to remove the salts by filtration, and the solvent was evaporated. This procedure was repeated twice. Hexane (30 mL) was then added, and the resultant solution was washed thrice with HCl aqueous solution (0.2 mM, 200 mL) and twice with NaCl solution (1 M, 200 mL). Magnesium sulfate was added at room temperature for 1 h, removed by filtration, and the resultant solution was evaporated and dried *in vacuo* to obtain the initiator as a white powder (yield: 80%).

MPC was polymerized at the initiator to obtain four types of PMPC-lipids with different degrees of polymerization (DP = 10, 20, 50, and 100). MPC (295 mg) was dissolved in a solvent mixture of methanol and dioxane (2:1 v/v, 2.1 mL). Ascorbic acid (10:17.6 mg, 20:8.8 mg, 50:3.5 mg, 100:1.8 mg), TPMA (10:2.9 mg, 20:1.45 mg, 50:0.58 mg, 100:0.29 mg), and copper bromide (10:0.22 mg, 20:0.11 mg, 50:0.045 mg, 100:0.022 mg) were added. Finally, the initiator (10:70 mg, 20:35 mg, 50:14 mg, 100:7 mg) was added and stirred for 24 h at 40°C. This solution was bubbled with nitrogen gas for 5 min before and after the addition of the initiator. For the initiator and MPC: [MPC]/[initiator] = 1/0.1 (MPC10-lipid), 0.05 (MPC20-lipid), 0.02 (MPC50-lipid), 0.01 (MPC100-lipid). The molar ratio of the initiator, ascorbic acid, TPMA, and copper bromide was [initiator]/[ascorbic acid]/[TPMA]/[CuBr_2_] = 1/1/0.1/0.01. The resultant solution was dropped into diethyl ether, and the supernatant was removed by decantation, followed by drying *in vacuo* for 3 h. The white powder was dissolved in pure water and dialyzed against pure water (molecular weight cutoff: 3.5 kDa) for 2 days. PMPC-lipids with DP of 10, 20, 50, and 100 were obtained as white powders via lyophilization (yield: MPC10-lipid: 92%, MPC20-lipid: 95%, MPC50-lipid: 96%, MPC100-lipid: 97%).

#### 2.2.2. Characterization of PMPC-lipids

##### 2.2.2.1. Gel permeation chromatography (GPC)

PMPC-lipids were dissolved in 70% methanol aqueous solution (1 mg/mL). The molecular weights of the polymers were determined using GPC (EXTREMA 4500Model; JASCO Co., Tokyo, Japan). The sample was filtered with a 0.22-µm filter. The column was SB-803-HQ (Shodex, Tokyo, Japan), and the development solution was 70% methanol aqueous solution. The measurements were performed at a flow rate of 0.50 mL/min at 25°C. PEG (Calibration Kit PEG-10; Agilent, Santa Clara, California, USA) was used for the calibration curve to determine the molecular weight.

##### 2.2.2.2. ^1^H-nuclear magnetic resonance (NMR) spectroscopy

The initiator was dissolved in chloroform-D (10 mg/650 µL), and PMPC-lipids were dissolved in methanol-D4 (10 mg/650 µL) for ^1^H-NMR spectroscopy (AVANCE 400; Bruker, Billerica, Massachusetts, USA). The analysis was performed using TopSpin 4.1.3. Lipid initiator: δ 0.87 (t, 6 H (-CH_3_)), 1.25 (s, 48 H (-CH_2_)_2_-), 1.61 (s, 4 H -(CH_2_)_2_-C-C(=O)-O-), 1.92 (s, 6 H (-C(Br)-CH_3_)), 2.31 (m, 4 H (-(CH_2_)_2_-C(=O)-)), 4.15–4.41 (m, 4 H (-O-CH_2_-)), 5.32 (m, 1 H (O-CH(C)-C). MPC10-lipid: δ 1.25 (s, 48 H (-CH_2_)_2_-), DPPE), 3.31 (s, 90 H -N+(CH_3_)_3_), 3.76 (br, 20 H -N-CH_2_-); MPC20-lipid: δ 1.26 (s, 48 H (-CH_2_)_2_-), DPPE), 3.31 (s, 180 H -N+(CH_3_)_3_), 3.76 (br, 40 H -N-CH_2_-); MPC50-lipid: δ 1.26 (s, 48 H (-CH_2_)_2_-), DPPE), 3.31 (s, 450 H -N+(CH_3_)_3_), 3.76 (br, 100 H -N-CH_2_-); MPC100-lipid: δ 1.26 (s, 48 H (-CH_2_)_2_-), DPPE), 3.31 (s, 900 H -N+(CH_3_)_3_), 3.77 (br, 200 H -N-CH_2_-) (Fig. S1).

##### 2.2.2.3. Critical micelle concentration (CMC) of PMPC-lipids

DPH was used to determine the CMC of PMPC-lipids. PMPC-lipids were dissolved in PBS (10 to 1.0 × 10^−5^ mg/mL), and DPH was dissolved in THF (30 µM). A solution of DPH (2 µL) was added to the PMPC-lipid solution (1 mL) and incubated at 37°C for 1 h. The fluorescence intensity of the resulting solution was measured using a spectrofluorometer (Ex: 357 nm, Em: 430 nm; FP-6500; JASCO).

##### 2.2.2.4. Characterization of PMPC-lipid micelles

The diameter and PDI of PMPC-lipids (1 mg/mL, in PBS) and zeta potential of PMPC-lipids (0.8 mg/mL, 1 mM NaCl aqueous solution) were measured via dynamic light scattering (DLS) using a Zetasizer Nano ZS (Malvern Instruments Co., Ltd, Worcestershire, UK).

### 2.3. Preparation of PMPC-lipid-modified liposomes

#### 2.3.1. Surface modification of liposomes via the external addition of PMPC-lipids

Liposomes were prepared using the thin-film hydration method. DPPC (1 mL, 10 mg/mL, in ethanol) and cholesterol (530 µL, 10 mg/mL, in ethanol) were mixed ([DPPC]/[cholesterol] = 1/1, by molar ratio), and the solution was evaporated to form a uniform lipid film, followed by drying *in vacuo* for 24 h. After the lipid film was hydrated in PBS (1.53 mL) using a magnetic stirrer for 2 h at room temperature, the liposome suspension was extruded through polycarbonate membranes (pore sizes: 1000, 400, 200, and 100 nm, nuclear track-etched membrane; Whatman, Ltd, UK). Extrusion was repeated at least 21 times using an Avanti Mini Extruder (Avanti Polar Lipids). To prepare fluorescently labeled liposomes, CF (1 mM, 1.53 mL) was used for extrusion. The resultant liposome suspension was washed by centrifugation (at 20,000 × *g* for 70 min at 4°C) to remove unencapsulated CF. This procedure was repeated twice. Finally, the liposome pellet was resuspended in 1 mL PBS to prepare liposomes encapsulating CF.

Liposome suspension (100 µL) was centrifuged at 20,000 × *g* for 70 min at 4°C, and the supernatant was completely removed. Then, a solution of PMPC-lipids (250 µL, 0.3 mM in PBS) was mixed with the liposome pellet and incubated for 30 min at 37°C. Next, cold PBS (1 mL) was added for centrifugation (at 20,000 × *g* for 70 min at 4°C) to remove the unbound PMPC-lipids. This procedure was repeated twice. Finally, the resultant liposomes were resuspended in PBS (100 µL) to prepare PMPC-lipid-modified liposomes.

#### 2.3.2. Characterization of PMPC-lipid-modified liposomes

##### 2.3.2.1. Measurement of the diameter and surface charge of PMPC-lipid-modified liposomes

The diameter and PDI of liposomes (1 mg/mL in PBS) and zeta potential of liposomes (0.8 mg/mL, 1 mM NaCl aqueous solution) were measured via DLS using Zetasizer Nano ZS. Modified liposomes were stored in PBS at 37°C for 14 d for DLS analysis.

#### 2.3.3. Transmission electron microscopy (TEM)

Modified liposomes were prepared using pure water instead of PBS as described above. Ammonium molybdate solution (2% in pure water, 10 µL) was mixed with the liposome suspension (1 mg/mL, 10 µL). The liposome suspension (10 µL) was placed on formvar/carbon-coated mesh grids for 1 min. The solution was then removed using filter paper, and the surface was dried with nitrogen gas. The samples were observed using JEM-2100 TEM (JEOL Ltd., Tokyo, Japan) at an acceleration voltage of 100 kV.

### 2.4. Evaluation of PMPC-lipid-modified liposomes

#### 2.4.1. Determination of cholesterol concentration in liposomes

The total cholesterol concentration in the liposomes was determined using a cholesterol kit (LabAssay Cholesterol; Fujifilm, Tokyo, Japan), according to the manufacturer’s instructions. Standard samples with cholesterol concentrations of 0, 0.5, 1, 2, 4, and 6 mg/mL were prepared using the cholesterol standard solutions. The liposome suspension was mixed with SDS solution (1 mg/mL, in MilliQ) and incubated for 15 min at 95°C to solubilize the liposomes. Then, a chromogenic reagent (3 mL) was added to the liposome suspension (40 µL) and incubated for 5 min at 37°C. Absorbance was measured at 600 nm using a multi-label plate reader (Wallac 1420 ARVOsx; PerkinElmer, Inc., Waltham, Massachusetts, USA).

#### 2.4.2. Phosphorus quantification

PMPC-lipid-modified liposomes were prepared using TBS (0.05 M, pH 7.6) instead of PBS as described above. Here, MPC10-lipids, MPC20-lipids, MPC50-lipids, and MPC100-lipids were used for modification. The liposomes were incubated at 37°C for 14 d. During incubation, liposomes were collected for the determination of phosphorus and cholesterol concentrations. The collected liposomes were centrifuged (at 20,000 × *g*, 70 min, 4°C) and adjusted to 1 mM cholesterol using TBS. The liposome suspension (100 µL) was mixed with sulfuric acid (10 N, 500 µL) and incubated at 160°C for 3 h. Then, hydrogen peroxide (10 µL) was added and incubated for 1.5 h, and the resulting solution was cooled to room temperature. Ammonium molybdate tetrahydrate aqueous solution (0.22%, 4.6 mL) and Fiske – SubbaRow reagent (200 µL) were added. The Fiske – SubbaRow reagent was composed of sodium hydrogen sulfite (15 g), sodium sulfite (0.5 g), and 1-amino-2-naphthol-4-sulfonic acid (0.25 g) in pure water (100 mL). The mixed solution was incubated at 100°C for 10 min, followed by measurement of the absorbance at 830 nm (ultraviolet-visible spectrophotometer, V-560; JASCO Co). The modified amount of PMPC-lipid was calculated as the difference between the phosphorus concentration of liposomes and that of PMPC-lipid-modified liposomes.

#### 2.4.3. GPC analysis of PMPC-lipid-modified liposomes

During incubation of the liposomes at 37°C for 14 d, the liposomes were analyzed via GPC (EXTREMA 4500Model; JASCO Co.) to measure the detached PMPC-lipids. The column was SB-803-HQ (Shodex), and the solution was PBS. The measurements were performed at a flow rate of 0.50 mL/min at 25°C.

#### 2.4.4. Analysis of adsorbed proteins onto liposomes in FBS

Liposome suspensions (100 µL) were incubated in 100% FBS (500 µL) for 1 h at 37°C. PBS (1 mL) was added and centrifuged at 20,000 × *g* for 70 min at 4°C. The supernatant was removed and resuspended in PBS. This procedure was repeated thrice to completely remove the unbound proteins from the liposomes. Finally, the liposome pellets were resuspended in PBS to a final cholesterol concentration of 4 mM. The diameter and PDI of liposomes were measured via DLS using Zetasizer Nano ZS.

The total protein concentration adsorbed on the liposomes incubated in FBS was determined using a Micro BCA Protein Assay Kit (Thermo Fisher Scientific, Waltham, Massachusetts, USA), according to the manufacturer’s instructions. Absorbance was measured at 562 nm using a multilabel plate reader. The total protein concentration adsorbed on the liposomes incubated in FBS was calculated by subtracting the liposomes treated with PBS from each sample.

The proteins adsorbed onto the liposomes were analyzed via SDS-polyacrylamide gel electrophoresis (PAGE) using the two gel solutions (5% and 12%). Liposome suspensions (4 mM, 18 µL) were added to SDS sample buffer (Tris-HCl [0.6 mL], 50% glycerol [5 mL], 10% SDS [2 mL], 2-mercaptoethanol [10.5 mL], 1% bromophenol blue [1 mL], MilliQ [0.9 mL]) and incubated at 99°C for 10 min. Samples (22 µL) were loaded onto the gel and electrophoresed at 40 V for 30 min and at 60 V for 2 h. Quick-CBB (Fujifilm Wako Pure Chemicals, Tokyo, Japan) was used for staining. The molecular weights of the proteins were determined by comparison with the Precision Plus Protein Standards (Bio-Rad, Hercules, California, USA).

#### 2.4.5. Analysis of encapsulation efficiency by liposome modification with PMPC-lipids

We compared the encapsulation efficiency with premixed PMPC-lipid modified liposome using FITC-albumin for this study. Liposome encapsulating FITC-albumin was prepared as described above, except CF. Briefly, DPPC (1 mL, 10 mg/mL, in ethanol) and cholesterol (530 µL, 10 mg/mL, in ethanol) were mixed, and the solution was evaporated to form a uniform lipid film, followed by drying *in vacuo* for 24 h. After the lipid film was hydrated in FITC-albumin solution (10 mg/mL, 1.53 mL in PBS) for 2 h at room temperature, the liposome suspension was extruded. The resultant liposome suspension was washed by centrifugation (at 20,000 × *g* for 70 min at 4°C) to remove unencapsulated FITC-albumin. Then, a solution of MPC100-lipid (250 µL, 0.3 mM in PBS) was mixed with the liposome pellet and incubated for 30 min at 37°C. Next, cold PBS (1 mL) was added for centrifugation (at 20,000 *× g* for 70 min at 4°C) to remove the unbound PMPC-lipids. This procedure was repeated twice. Finally, the resultant liposomes were resuspended in PBS (1000 µL). On the other hand, premixed PMPC-lipid modified liposome was prepared by mixing DPPC (1 mL, 10 mg/mL, in ethanol), cholesterol (530 µL, 10 mg/mL, in ethanol) and MPC100-lipid (824 µL,10 mg/mL, in ethanol) ([DPPC]/[cholesterol]/[PMPC-lipid] = 1/1/0.02), followed by same procedure for liposome preparation using FITC-albumin solution (10 mg/mL, 1.53 mL in PBS). Those two liposome suspensions were mixed with SDS solution (1 mg/mL, in MilliQ) and incubated for 15 min at 95°C to solubilize the liposomes. The liposome concentration was then adjusted to 2 mM, and the fluorescence intensity of the resulting solution was measured using a spectrofluorometer (Ex: 488 nm, Em: 516 nm; FP-6500; JASCO) to measure the encapsulated FITC-albumin in the liposome.

#### 2.4.6. Analysis of the interactions between PMPC-modified liposomes and anti-PEG antibodies using flow cytometry

Latex beads (40 mg/mL, 250 µL, aldehyde/sulfate latex, diameter: 4 µm) were mixed with PBS (500 µL) and centrifuged at 3,000 × *g* for 20 min at 4°C. The supernatant was removed, and the cells were resuspended in PBS. This procedure was repeated twice. Finally, the latex beads were resuspended in 500 µL PBS. Next, anti-PEG antibody (1 mg/mL, 10 µL) was added to the latex bead solution (20 mg/mL, 100 µL) and incubated for 24 h at 4°C. After the incubation, the bead suspension was centrifuged at 3,000 × *g* for 20 min at 4°C. The supernatant was removed, and the cells were resuspended in PBS. This procedure was repeated thrice to remove the unreacted anti-PEG antibody. Finally, anti-PEG-antibody-conjugated beads (10 mg/mL) were resuspended in BSA solution (1 mg/mL, 200 µL, in PBS). In this experiment, we used fluorescently labeled liposomes (1 mM cholesterol) for modification with PMPC-lipids and PEG-lipid. Anti-PEG antibody-beads (10 mg/mL, 5 µL in BSA) were added to modified liposome suspension (100 µL) and incubated for 30 min at 37°C. After incubation, the bead suspension was centrifuged at 3,000 × *g* for 5 min at 4°C to remove the unbound liposomes. The liposomes were resuspended in PBS (100 µL). The suspension was passed through a cell strainer and measured via flow cytometry (GALLIOS, Beckman Coulter, Inc., California, USA).

### 2.5. Statistical analysis

All experiments were repeated at least thrice. Data are presented as the mean ± standard deviation or representative images. Statistical analysis was conducted using a one-way analysis of variance, followed by Dunnett’s multiple comparison tests with a single pooled variance using GraphPad Prism 9 for MacOS version 9.0.0 (GraphPad Software, La Jolla, California, USA). The calculated p-values are defined as follows: *p < 0.05, **p < 0.001, ***p < 0.001, and ****p < 0.0001.

## 3. Results and discussion

### 3.1. Characterization of PMPC-lipids

**Figure 1.**
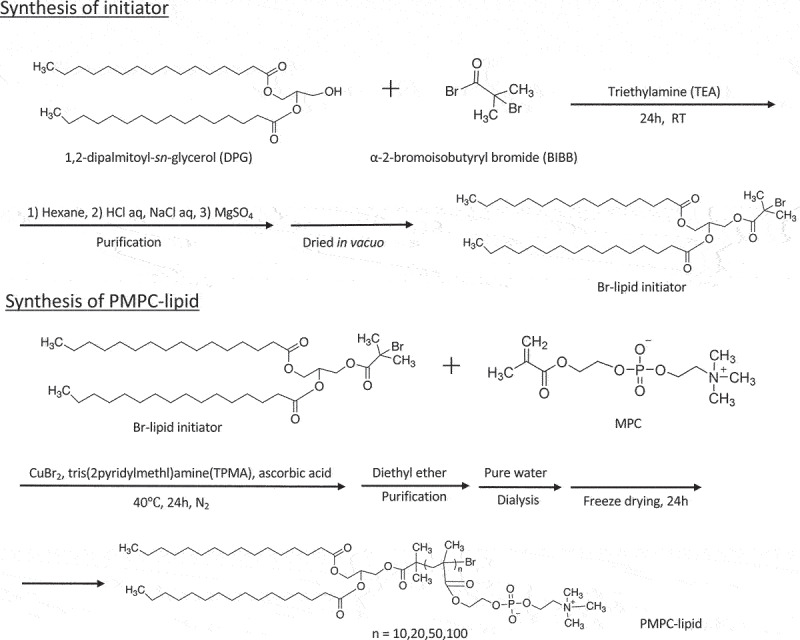
Schematic overview of poly(2-methacryloyloxyethyl phosphorylcholine) (polyMPC)-conjugated lipid (PMPC-lipid) synthesis. MPC was polymerized at the lipid initiator using the activators regenerated by electron transfer-atom transfer radical polymerization (ARGET-ATRP) method with different degrees of polymerization from 10–100. MPC; 2-methacryloyloxyethyl phosphorylcholine, ARGET-ATRP; activators regenerated by electron transfer-atom transfer radical polymerization.

PMPC-lipids with DP of 10, 20, 50, and 100 (MPC10-lipid, MPC20-lipid, MPC50-lipid, and MPC100-lipid, respectively) were synthesized using the activators regenerated by electron transfer (ARGET)-ATRP method (Figure 1). The molecular weights were calculated via ^1^H-NMR and GPC analyses and were consistent with the theoretical values. All PMPC-lipids had CMC, indicating amphiphilicity (Table 1). Moreover, CMC increased with an increase in DP owing to an increase in the hydrophilic part. DLS analysis showed that the diameter of PMPC-lipid micelles increased with an increase in PMPC units, which also supported the successful synthesis of PMPC-lipids. The zeta potential of the micelles differed among various PMPC-lipids, where MPC100-lipid micelles were more neutral than shorter PMPC-lipids, indicating the shielding effect of the MPC polymer. The micelles showed negative charge of the zeta potential although the MPC unit is neutral. Presumably, hydrophobic core influences the surface charge of the micelles although the mechanism is unclear. Taken together, we successfully synthesized PMPC-lipids with different DP of the PMPC segment.

**Table 1.** Characterization of poly(2-methacryloyloxyethyl phosphorylcholine) (polyMPC)-conjugated lipids (PMPC-lipids).

PMPC-lipid	Mw (theoretical value)	Mw (measured value)	Degree of polymerization	Mw/Mn	CMC [mg/mL]	Micelle diameter^a^ [nm]	PDI [-]	Zeta potential [mV]
MPC10	3.7 × 10^3^	4.3 × 10^3^	12	1.2	3.5 × 10^−3^	5.2 ± 0.8	0.99 ± 0.02	−12.0 ± 1.2
MPC20	6.6 × 10^3^	7.8 × 10^3^	24	1.2	3.5 × 10^−2^	7.9 ± 1.4	0.83 ± 0.08	−6.8 ± 1.6
MPC50	1.5 × 10^4^	1.6 × 10^4^	52	1.3	2.5 × 10^−1^	8.9 ± 0.9	0.75 ± 0.04	−5.4 ± 1.6
MPC100	3.0 × 10^4^	2.9 × 10^4^	94	1.3	4.5 × 10^−1^	13.0 ± 1.7	0.50 ± 0.08	−3.8 ± 0.4

### 3.2. Evaluation of PMPC-lipid-modified liposomes

**Figure 2.**
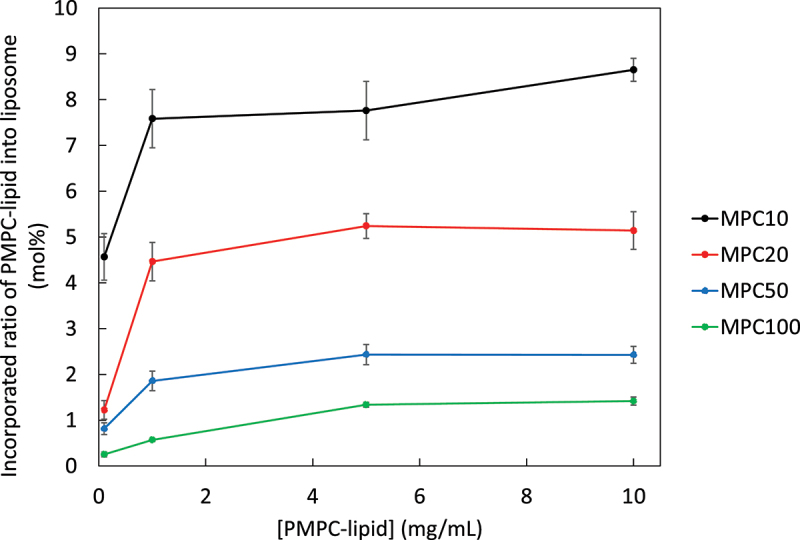
Surface modification of liposomes via the exogenous addition PMPC-lipids. Relationship between incorporated PMPC-lipid ratio into liposomes and adding concentration. Incorporated PMPC-lipids were analyzed via phosphorus quantification by comparing with non-modified liposomes.

We attempted to modify the liposome surface by exogenously adding PMPC-lipids, which would be incorporated into the lipid bilayer membrane via hydrophobic interaction-driven self-assembly. Here, we incubated PMPC-lipids with liposomes for 30 min at 37°C and examined the influence of DP (from 10 to 100) on the incorporation by measuring phosphorus concentration (Figure 2). The incorporated ratio of PMPC-lipid increased with an increase in the feed concentration and reached a plateau at a certain concentration, although the incorporation ratio differed among different PMPC-lipids. The maximum incorporated ratio of MPC10-lipid, MPC20-lipid, MPC50-lipid and MPC100-lipid was 9.5, 5.7, 2.3, and 1.7 mol%, respectively, indicating that more PMPC-lipids were incorporated into the liposome as the DP of PMPC segment became smaller. Since the feed concentrations are higher than the CMC, PMPC-lipids form micelles, and monomeric PMPC-lipids can be incorporated into the liposome surface under equilibrium. Exogenous incorporation was influenced by the excluded volume of PMPC, where the larger size of PMPC-lipid hindered the incorporation.

**Figure 3.**
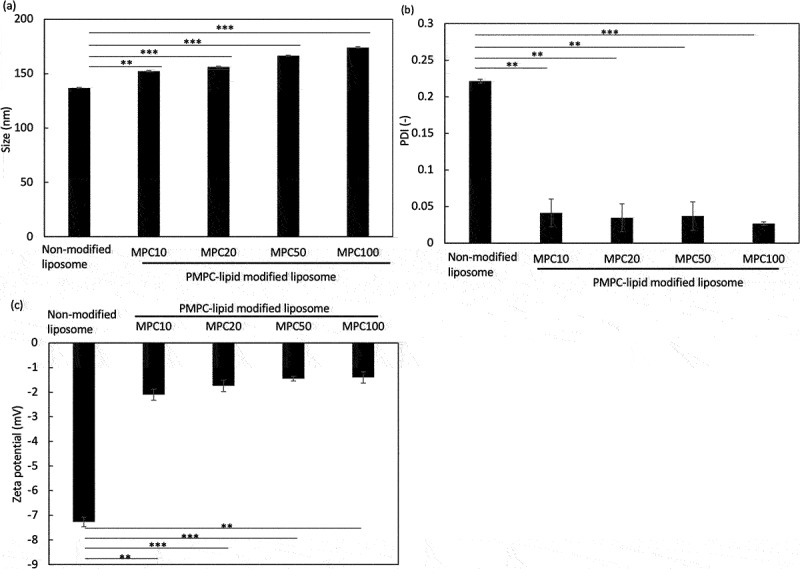
Dynamic light scattering (DLS) analysis of PMPC-lipid-modified liposomes by measuring (a) Size, (b) polydispersity index (PDI), and (c) zeta potential. Non-modified liposomes were analyzed as controls.

After liposomes were treated with each PMPC-lipid, we analyzed PMPC-lipid-modified liposomes using the DLS method (Figure 3(a): particle diameter, Figure 3(b): PDI, Figure 3(c): zeta potential). The sizes of PMPC-lipid-modified liposomes were 152 ± 1 nm (MPC10-lipid), 156 ± 1 nm (MPC20-lipid), 167 ± 1 nm (MPC50-lipid), and 174 ± 1 nm (MPC100-lipid), whereas the size of unmodified liposomes was 137 ± 1 nm (Figure 3(a)), indicating that PMPC-lipid was incorporated into the liposome surface and the particle diameter increased with an increase in the DP of PMPC-lipids. The PDI of modified liposomes significantly reduced when they were treated with PMPC-lipids (0.22 ± 0.01 [non-modified], 0.041 ± 0.019 [MPC10-lipid], 0.035 ± 0.019 [MPC20-lipid], 0.037 ± 0.019 [MPC50-lipid], and 0.027 ± 0.003 [MPC100-lipid]), indicating that liposomes were dissociated from the aggregation state in non-modified liposomes by the spontaneous surface modification with PMPC-lipids (Figure 3(b)). No difference in PDI was observed among the different PMPC-lipid-modified liposomes. The zeta potential of modified liposomes also proved that the liposome surface was covered with the PMPC layer, since the zeta potential of modified surfaces shifted to more neutral charge compared to non-modified liposomes (−2.1 ± 0.2 mV [MPC10-lipid], −1.7 ± 0.2 mV [MPC20-lipid], −1.5 ± 0.1 mV [MPC50-lipid], and −1.4 ± 0.2 mV [MPC100-lipid], −7.3 ± 0.2 mV [non-modified]) (Figure 3(c)), where no significant difference was observed among PMPC-lipid groups.

**Figure 4.**
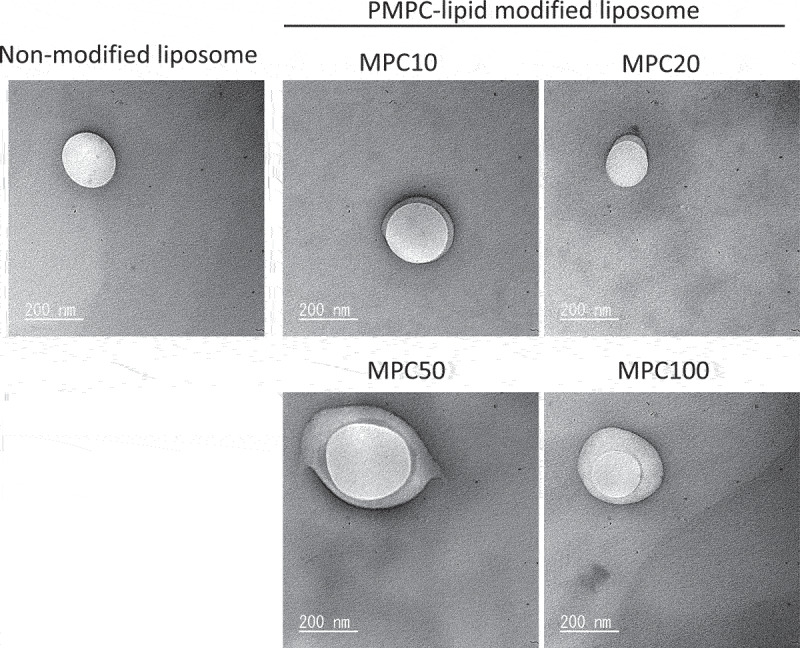
Representative transmission electron microscopy (TEM) images of non-modified and PMPC-lipid-modified liposomes. The sample was treated with 2% ammonium molybdate solution for negative staining and TEM observation.

**Figure 5.**
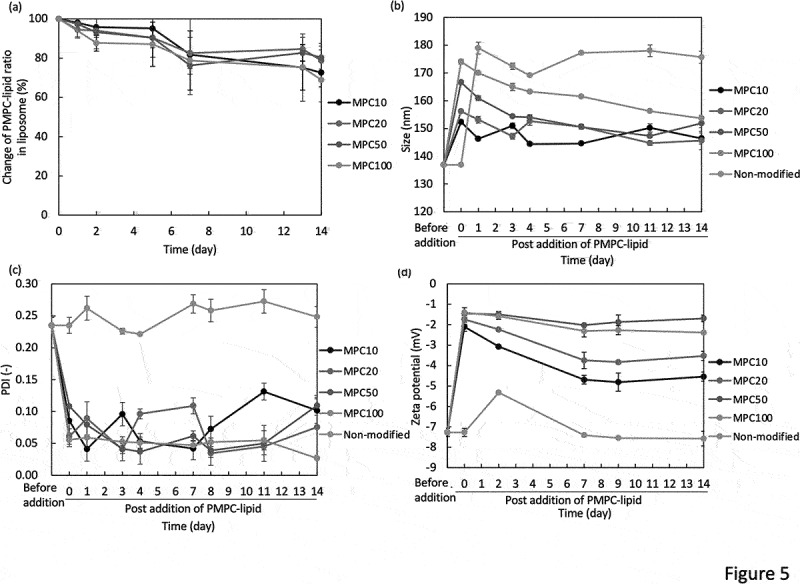
Evaluation of the long-term stability of PMPC-lipid-modified liposomes by measuring the (a) PMPC-lipid ratio via phosphorus quantification, and DLS-analysis of their (b) size, (c) PDI, and (d) zeta potential. All the liposomes were stored at 37°C. Prepared liposomes before mixing with PMPC-lipids are indicated as Before addition. As a control, non-modified liposome was used, and there was some aggregation and precipitates observed during the storage period.

**Figure 6.**
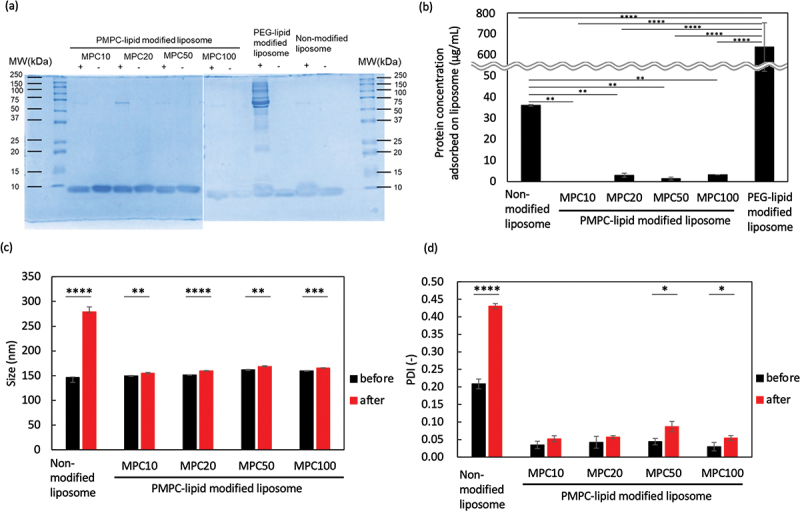
Analysis of modified liposomes in FBS. The adsorbed serum proteins on liposomes was analyzed via (a) sodium dodecyl sulfate-polyacrylamide gel electrophoresis (SDS-PAGE), (b) determination of total protein concentration, and (c, d) DLS ((c) particle size and (d) PDI). (a) is representative picture of SDS-PAGE gels after Coomassie Brilliant Blue staining of proteins adsorbed to liposomes modified with PMPC-lipids after incubation in fetal bovine serum (FBS). Lane 1: ladder 10–250 kDa; Lane 2,3: MPC10-lipid-liposomes; Lane 4,5: MPC20-lipid-liposomes; Lane 6,7: MPC50-lipid-liposomes; Lane 8,9: MPC100-lipid-liposomes; Lane 10,11: PEG-lipid-liposomes; Lane 12, 13: non-modified liposomes. + indicates the presence of FBS and – indicates absence of FBS.

We also observed the liposomes using TEM (Figure 4). Both unmodified and modified liposomes were spherical in shape. Notably, we observed a thick polymer layer surrounding the liposomes, indicating surface modification with PMPC-lipids. We could observe most likely remaining liposomes, most of which were detached from the surface while washing out, and also were destroyed when the liposomes were observed under dry and vacuum conditions. In addition, it did not look as if the observed MPC polymer layers covered the liposome surface homogeneously. We assumed that all the samples were observed under dried and vacuum conditions, so that the morphology of MPC polymer layers was different from that in the solution state. However, the whole liposome surface was covered with MPC polymers as interpreted from the results of the subsequent stability tests (Figure 5, Supplementary Fig. S3) and protein-binding tests (Figure 6). Thus, we demonstrated that the liposome surface could be modified with amphiphilic PMPC-lipids via spontaneous incorporation.

We also studied the influence of the inner space by the PMPC polymer layer on the encapsulation of active pharmaceutical ingredients. Here, we used FITC-albumin as a model for active pharmaceutical ingredients. When we compared the encapsulated FITC-albumin in exogenously modified liposome with premixed liposome, the exogenously modified liposome showed higher efficiency (3.1 times higher concentration, Supplementary Fig. S2). This result indicated that inner space of non-modified liposome is more available for the loading of albumin than pre-modified liposome. Therefore, our exogenous modification with PMPC-lipids is useful for the highly efficient loading of active pharmaceutical ingredients.

### 3.3. Long-term stability of PMPC-lipid-modified liposomes

The stability of PMPC-lipid-modified liposomes was evaluated for 14 d by measuring the incorporated ratio of PMPC-lipids, liposome size, PDI, and zeta potential (storage temperature: 37°C for Figures 5 and 4 °C for Supplementary Fig. S3). PMPC-lipid-modified liposomes were incubated at 37°C, and detached PMPC-lipids were measured over time via phosphorus quantification (Figure 5(a)). As a control, we incubated non-modified liposomes. During the storage, the non-modified liposome showed the aggregation and some precipitation. On the other hand, all the modified liposomes did not show any aggregation during the storage period, indicating the effect of the surface modification with PMPC-lipids.

We found that PMPC-lipids gradually detached over time, and approximately 20–30% of free PMPC-lipids were detected in the fluid phase after 14 d at 37°C, while almost no detachment of PMPC-lipids was observed for 14d when stored at 4°C (Supplementary Fig. S3). There was no significant difference in the detachment rate among the different DP of PMPC-lipids when stored at 37°C. There was also a similar tendency for changes in the particle size of the modified liposomes. The particle size gradually decreased over 14 d (Figure 5(b)) and was closer to the original liposome size; in particular, the liposomes modified with MPC50-lipid and MPC100-lipid showed a larger reduction in size (20 nm), which was reflected by the detachment of the PMPC-lipid from the liposome surface. In addition, the change in zeta potential was consistent with the change in particle size (Figure 5(d)), where the zeta potential was shifted to a negative charge, particularly for liposomes modified with MPC50-lipid and MPC100-lipid. In contrast, PDI did not change significantly for any of the modified liposomes (Figure 5(c)), indicating that the suspension dispersity was well maintained over time. Regarding the modified liposomes stored at 4°C, almost no significant change in liposome size, PDI and zeta potential was observed for 14 d (Supplementary Fig. S3). These results suggest that the surface modification effect was well maintained over 14 d in terms of suspension dispersity, although some PMPC-lipids were detached from the surface. The two palmitoyl chains in the hydrophobic region were sufficient to anchor the PMPC segment via hydrophobic interactions.

### 3.4. Effect of PMPC-lipid modifications on the interactions between blood proteins and anti-PEG antibodies

After we incubated the modified liposomes in 100% FBS for 1 h, the adsorbed blood proteins were analyzed by SDS-PAGE (Figure 6(a)) and measuring the total protein concentration (Figure 6(b)), followed by DLS analysis (Figure 6(c): particle diameter, Figure 6(d): PDI). Protein adsorption was significantly reduced in the PMPC-lipid-modified liposomes (MPC10: 0.0 ± 0.3 µg/mL, MPC20: 3.0 ± 1.9 µg/mL, MPC50: 1.4 ± 1.0 µg/mL, MPC100: 3.2 ± 0.7 µg/mL) compared to the unmodified liposomes (36.0 ± 1.6 µg/mL) and PEG-lipid-modified liposomes (637.9 ± 115.0 µg/mL), and no difference in protein adsorption was detected among different PMPC-lipids. SDS-PAGE analysis also revealed that almost no bands were detected for PMPC-lipids-modified liposomes, although a weak band at around 65 kDa (albumin) was detected, which is consistent with the total protein concentration determination results. On the other hand, there was lots of binding of proteins from serum for PEG-lipid-modified liposomes. In particular, a band at around 65 kDa (albumin) was strongly detected. We considered that the exogenous modification of liposome by adding PEG-lipid could not cover the whole liposome surface, so that albumin was bound onto the liposome surface. In fact, when we studied the protein binding onto PEG-lipid-modified liposomes from human plasma, where PEG-lipid was premixed with DPPC and cholesterol, the protein adsorption level was comparable to PMPC-lipid-modified liposomes [23].

When the liposomes were stored in 100% FBS, the particle diameter of the unmodified liposomes significantly increased, whereas the PMPC-lipid-modified liposomes had similar particle sizes although there was a significant small increase for all modified liposomes (approximately 5–8 nm increase). Regarding PDI, there was also a small increase seen for PMPC-lipid-modified liposomes, although unmodified liposomes showed a large increase in PDI (Figure 6(c,d)). Thus, exogenously modified PMPC-lipids can prevent protein adsorption in FBS onto the liposome surface and maintain their surface properties, indicating that PMPC-lipid-modified liposomes are stable in FBS without the strong aggregation.

The incorporated ratio of MPC100-lipid was 1.4 mol%, which was much lower than that of MPC10-lipid (8.7 mol%) and MPC20-lipid (5.1 mol%). However, no differences were observed in the protective effects on protein adsorption. Because the molecular weight of PMPC is different, each PMPC-lipid has a different excluded volume, so the incorporated ratio is different at the end, which limits the incorporated ratio. These results show that spontaneously incorporated PMPC-lipids can form a fully packed PMPC layer on the liposome surface and exert protective effects against protein adsorption.

**Figure 7.**
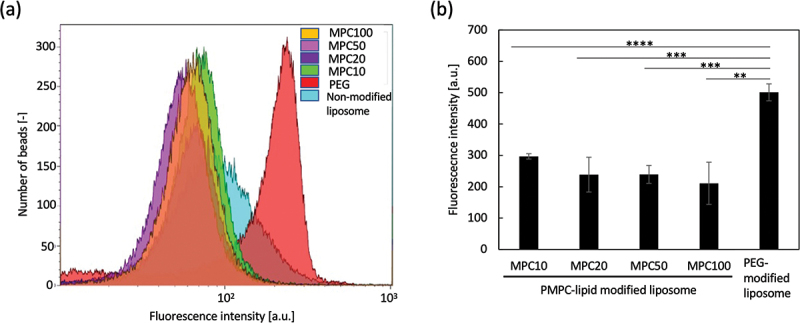
Flow cytometric analysis of the interactions between PMPC-lipid-modified liposomes and the anti-poly(ethylene glycol) (PEG) antibody. (a) Histogram plots and (b) mean fluorescence intensity of fluorescently labeled liposomes bound to the anti-PEG antibody-immobilized latex beads. PEG-lipid-modified and non-modified liposomes were used as controls. The background intensity from liposome was subtracted from each fluorescence intensity to calculate the mean fluorescence intensity.

Finally, we studied the interaction between PMPC-lipid-modified liposomes and anti-PEG antibodies using flow cytometry. Since the size of the modified liposomes was much smaller than that of the cells, it was impossible to directly detect the liposomes via flow cytometry. Therefore, we attempted to detect fluorescently labeled liposomes bound to anti-PEG antibody-immobilized beads (size: 4 µm). A fluorescence dye (CF) was encapsulated into liposomes, followed by modification with PMPC-lipids or PEG-lipids as controls, and they were treated with anti-PEG antibody beads for flow cytometry analysis (Figure 7). There was a strong binding of PEG-lipid-modified liposomes onto the beads, while no significant binding of any of the PMPC-lipid-modified liposomes was detected, indicating that there was no cross-reactivity between the anti-PEG antibody and PMPC-lipid-modified liposomes. Since the reaction mechanism of the anti-PEG antibody with PEG chains is still unclear, there is a possibility that the antibody cross-reacts with other synthetic polymers. The antibody normally recognizes the epitope of the PEG chain, which is composed of repeated units of ethylene glycol (-CH_2_-CH_2_-O-), and an epitope may be mimicked by PMPC. Therefore, it is important to compare the reactivity of anti-PEG antibodies between PEG-modified and PMPC-modified liposomes. This result suggests that PMPC-lipid-modified liposomes cannot be recognized by anti-PEG antibodies which already present in the recipient. Some studies have reported that healthy individuals who have not previously been administered PEGylated therapeutics already have anti-PEG antibodies as a consequence of previous exposure to PEG since the PEG is contained in food and cosmetics [18]. It is also known that some vaccines for coronavirus disease 2019 contain PEG-conjugated lipids [24,25] and in some cases have been reported to evoke an anaphylatoxic reaction [26,27]. Therefore, our PMPC-lipid can be used for the surface modification of drug carriers when the patients have already developed anti-PEG antibodies. From our in vitro study, we cannot see clear difference among liposomes modified with four different PMPC-lipids. Therefore, we will run in vivo study by injecting those modified liposomes into rodents and study the influence of different PMPC length on biodistribution, circulation time and inflammatory reactions. Previous reports on PEGylated liposomes showed that the molecular weight of PEG and type of lipid could influence on the circulation time [28,29]. For example, PEGylated liposomes with stearoyl acyl chains and 2 kDa PEG chain showed the longest circulating time. From our previous papers, it can be concluded that the longer PMPC chain showed more effective protection on protein adsorption in human blood [22,23]. Therefore, it will be important to optimize the molecular weight of PMPC and lipid length for prolonging the circulating time in vivo.

We are aware of the potential risk of an immune response against the PMPC-modified liposomes and will perform an in vivo analysis of antibody production. According to the other zwitterionic polymer case, there was a report that production of IgM, but not of IgG, was induced by injecting zwitterionic polycarboxybetaines (PCBs)-modified liposome in mice [30]. Therefore, not only PEG but also zwitterionic polymers can possibly induce antibody production in vivo. We will therefore study the immunological response in animal models.

## 4. Conclusions

Surface modification could be achieved by mixing PMPC-lipids with liposome, where inserted PMPC-lipids could prevent liposome aggregation and blood protein adsorption, regardless of the DP of PMPC. In addition, the modified liposome surface was stable, and the effect was kept during 14 days, indicating the long stability and shielding effect. With this surface modification technique, we could efficiently encapsulate water-soluble active pharmaceutical ingredients since the inner space is completely available for loading. Also, our PMPC-lipids could be alternative to PEG-lipid for the liposome coating since anti-PEG antibody could not recognize the polymer, so that they are available for patients with anaphylatoxic reaction against PEG. Thus, we successfully fabricated biologically inert liposome surfaces via spontaneous modifications with PMPC-lipids, which is promising for new liposome drugs.

## Supplementary Material

Supplemental Material

Supplemental Material

Supplemental Material

Supplemental Material
