# Supplementary material for: Impact of spontaneous liposome modification with phospholipid polymer-lipid conjugates on protein interactions
Source: Sci Technol Adv Mater. 2022 Dec 8;23(1):845–57. doi: 10.1080/14686996.2022.2146466 (PMC9744213; doi:10.1080/14686996.2022.2146466)
Supplement: Supplemental Material [file TSTA_A_2146466_SM4461.docx]

**Supplementary Figure S1.** ^1^H-NMR spectrum of PMPC-lipids.

**Supplementary Figure S2.** Influence of surface modification with PMPC-lipids on encapsulation efficiency. The fluorescence intensity of encapsulated FITC-albumin in modified liposomes, either which was premixed with MPC100-lipid or which was exogenously modified with MPC100-lipid, was compared to study the efficiency. The total lipid concentration was 2mM for the measurement.

**Supplementary Figure S3.** Evaluation of the long-term stability of PMPC-lipid-modified liposomes by measuring the (a) PMPC-lipid ratio via phosphorus quantification, and DLS-analysis of their (b) size, (c) PDI, and (d) zeta potential. All the liposomes were stored at 4 °C. Prepared liposomes before mixing with PMPC-lipids are indicated as Before addition.
