# Supplementary figures and images for: Impact of spontaneous liposome modification with phospholipid polymer-lipid conjugates on protein interactions
Source: Sci Technol Adv Mater. 2022 Dec 8;23(1):845–57. doi: 10.1080/14686996.2022.2146466 (PMC9744213; doi:10.1080/14686996.2022.2146466)

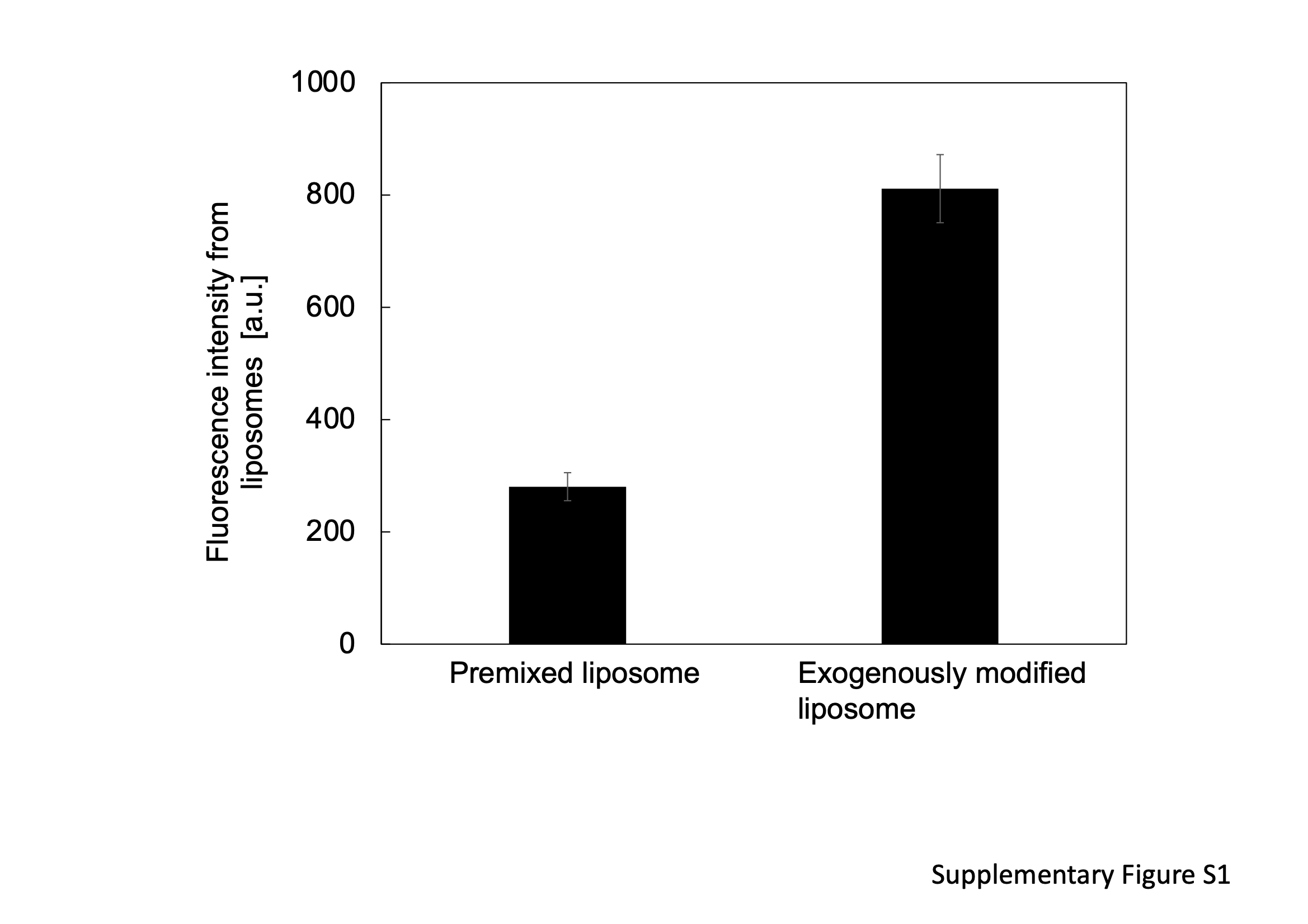

Supplement: Supplemental Material [file TSTA_A_2146466_SM4460.png]

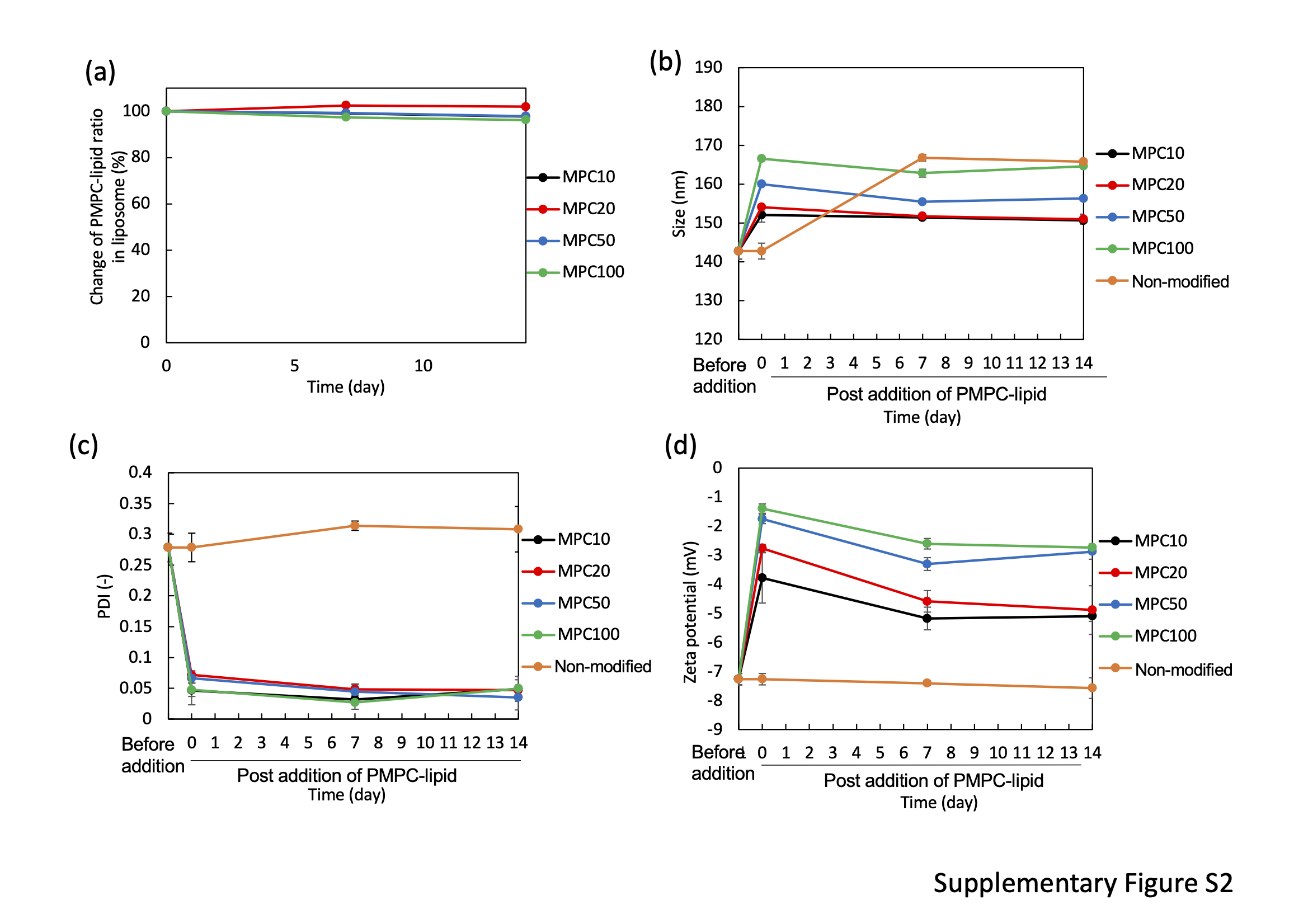

Supplement: Supplemental Material [file TSTA_A_2146466_SM4459.png]

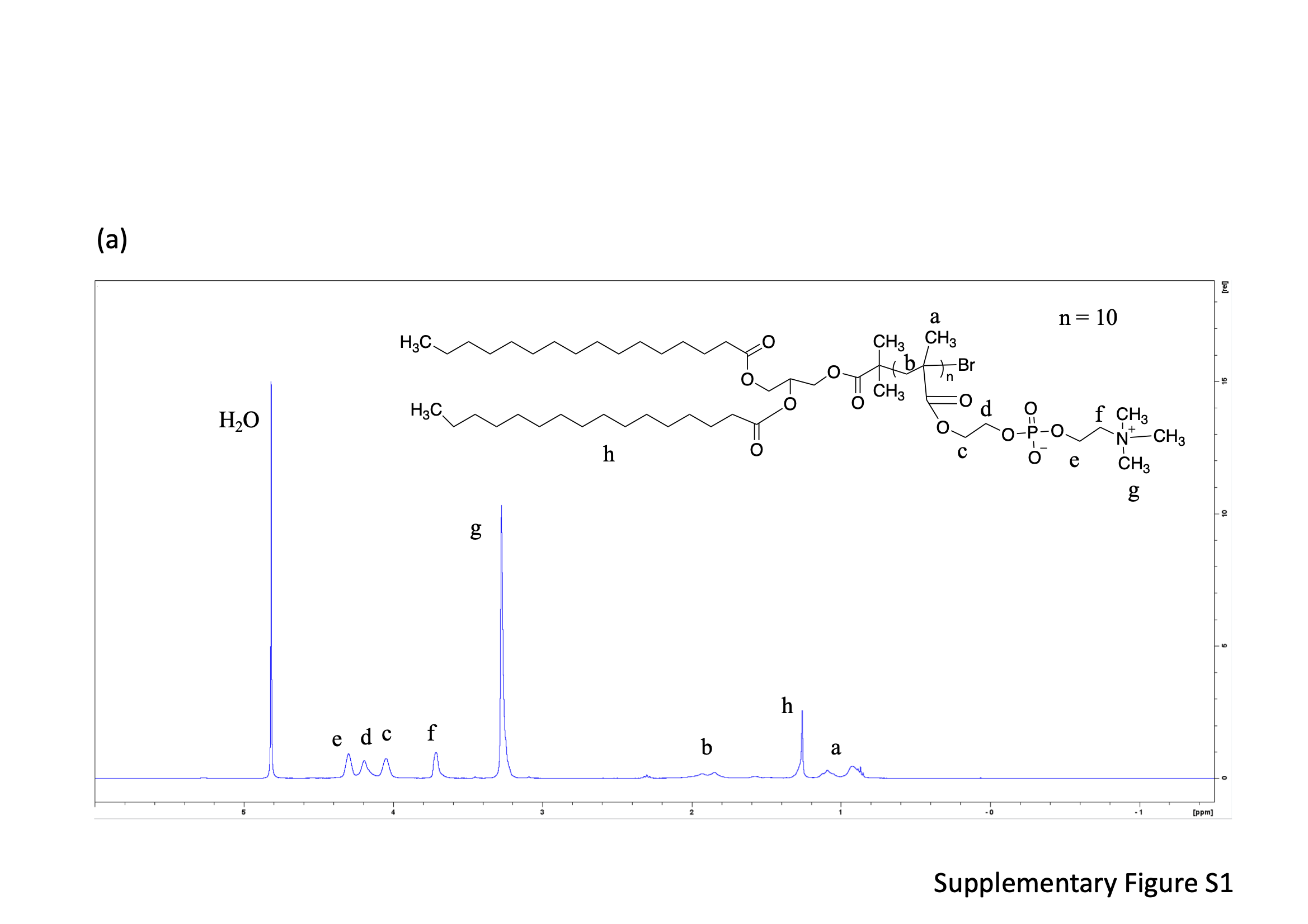

Supplement: Supplemental Material [file TSTA_A_2146466_SM4458.png]
